# Hand, foot and mouth disease - a short case report

**DOI:** 10.4317/jced.52031

**Published:** 2015-04-01

**Authors:** Roopashri-Rajesh Kashyap, Rajesh-Shanker Kashyap

**Affiliations:** 1 M.D.S. Reader, Dept. of Oral Medicine and Radiology, A.J. Institute of Dental Sciences, Mangalore- 575004, Karnataka, India; 2 M.D.S. Professor, Dept. of Periodontics, Yenepoya Dental College, Mangalore, Karnataka, India

## Abstract

Hand, foot and mouth disease, that was once considered a disease of cattle, has been emerging as a common human childhood disease in the last few years. It is a viral disease characterized by a brief febrile illness and typical vesicular rashes. In rare cases, patients may also develop neurological complications. This report describes a case of hand, foot and mouth disease, presented with typical clinical features in the South Indian region.

** Key words:**Hand, foot and mouth disease, viral lesions, blisters.

## Introduction

Hand, foot and mouth disease (HFMD) is a common childhood disease characterized by a brief febrile illness, typical vesicular rashes on the palms, soles, or buttocks, and oropharyngeal ulcers. In rare cases, patients may also develop neurological complications, such as encephalomyelitis, aseptic meningitis, and acute ﬂaccid paralysis. The most common etiological agents are coxsackievirus A6, A16 and enterovirus type 71 (1,2). Here we report a case of a two- year old child presented with typical features of HFMD.

## Case Report

A two years old boy reported to the hospital with the complaint of difficulty in eating since a day. History revealed the presence of fever since a day. The patient had developed fever suddenly the previous night without any symptoms of flu. After 12 hours his mother had noticed two papules; one in the palm and one in the foot. Following that he developed a severe ache in legs and difficulty to eat. The patient experienced a severe itching over the papules.

**Figure 1:**
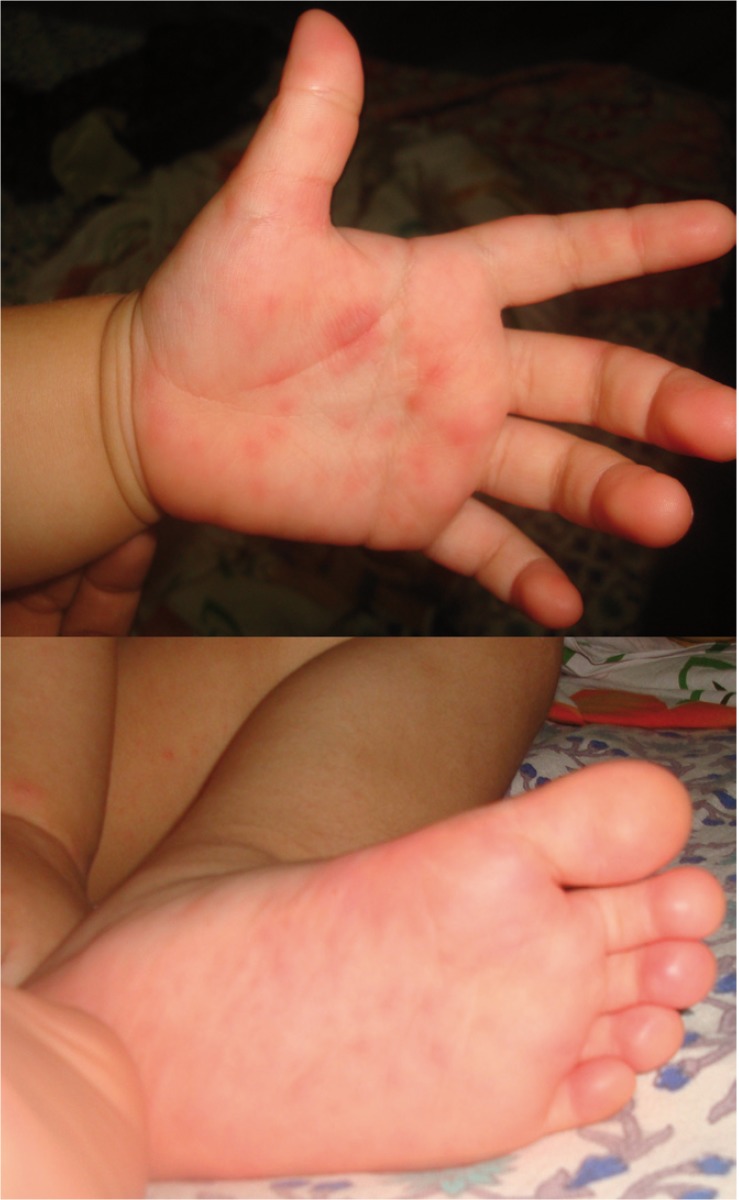
Papules on hands and feet on the first day.

**Figure 2:**
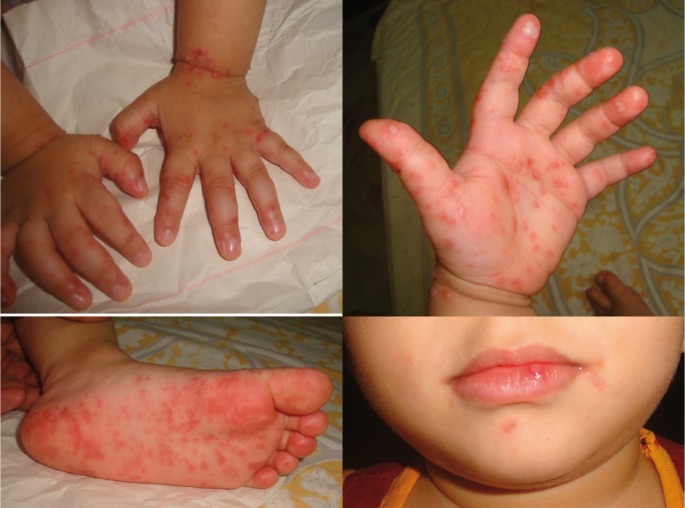
Papules and blisters on the hand, foot and mouth on the third day.

**Figure 3:**
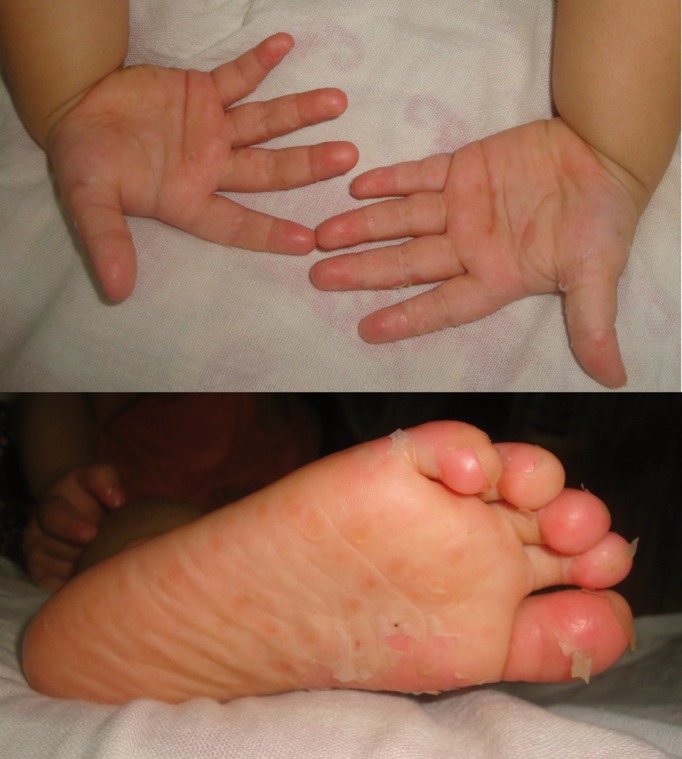
Healing lesions on hand and foot on the 15th day.

Informed consent was obtained from the parent as a part of the routine protocol before the clinical examination. On examination, the patient was febrile and had a body temperature of 101.4°F. Many papules were noted on the palm and foot. 2-3 papules were present over the trunk region and knee as well (Fig. 1). Intraoral examination revealed multiple reddish macules, measuring approximately 2mm in diameter in the roof of the hard palate. No other lesions were present intraorally. Based on the clinical features, the case was diagnosed as hand, foot and mouth disease. The patient was advised to consume plenty of fluids and was prescribed paracetamol syrup to control fever, topical local anesthetic for intraoral application, antihistamine syrup to reduce itching and calamine lotion for topical application. After two days, most of the papules had turned into fluid filled blisters and few blisters were present around the mouth (Fig. 2). However, palatal lesions had subsided improving the patient’s ability to take food. Vesicles started forming crustations in a week and the skin returned to normal in a month (Fig. 3). The patient was followed up for 6 months and no recurrence was noted.

## Discussion

HFMD is a common, usually mild childhood illness caused by enteroviruses. Over the last five years, coxsackievirus A6 has been identified as a causative agent in outbreaks in Europe, South-East Asia and America (3). HFMD was first reported in New Zealand in 1957. Coxsackievirus A16 was first identified next year in 1958 in Canada. HFMD has been considered to be a benign disease of self limiting nature (4). The term HFMD derives from typical maculopapular or vesicular lesions involving the skin of the hands, feet and oral mucosa. The prodromal phase, including low-grade fever, malaise and sore throat is commonly observed (5).

HFMD is characterized by the sudden appearance of erythematous papulo vesicular eruptions. Vesicles are round or oval. Generally, they appear in crops and persist in groups over some specific areas like hand, feet, perioral area, knees, buttocks and also intraorally. Lesions in thick skin like the palms and soles may not develop classical vesicle; they may instead persist as erythematous papules. The disease usually improves spontaneously after 7-10 days without any complication. In severe disease, cardiorespiratory and neurological involvement may develop.

HFMD is generally easily diagnosed on clinical grounds. Although, this shares some clinical resemblance with other diseases like varicella zoster, papular urticaria, impetigo and pompholyx, the constellation of features are unique enough to aid instant clinical diagnosis with certainty in almost all cases. There is neither an effective antiviral therapy, nor an effective vaccine available against the disease. This is a contagious disease and has the potentiality to spread very fast over a large population in the community (4). Treatment for HFMD is mainly symptomatic. Antipyretics are given to control fever and antihistamines to reduce itching. Topical local anesthetics can be prescribed for oral ulcers to improve patients’ ability to consume a routine diet.

Prevention of further spread of the disease is the only way to control a disease from becoming a large outbreak. As the organisms are enterovirus, they spread through faeco-oral route. Strict implementation of basic protocols like monitoring cleanliness of the hands, utensils and drinking water and avoiding direct contact with affected people can be rewarding. Restriction of the affected children from attending school or other outdoor activities is a very simple but effective strategy (4).

## Conclusions

HFMD, that was once considered a disease of cattle, has been emerging as a common human childhood disease in the last few years. The incidence of this disease increases every year. Though in most of the cases, it is nonfatal, there are some reported cases of complications seen in HFMD patients. All dentists, pediatricians and dermatologists should be aware of the clinical features of this disease and possible complications.
